# Preconception underweight impact on postnatal osteoporotic fracture: a retrospective cohort study using Japanese claims data

**DOI:** 10.1186/s12884-024-06514-y

**Published:** 2024-04-25

**Authors:** Kayoko Kaneko, Maiko Suto, Eiko Miyagawa, Masashi Mikami, Yukio Nakamura, Atsuko Murashima, Kenji Takehara

**Affiliations:** 1 Division of Maternal Medicine, Center for Maternal-Fetal, Neonatal and Reproductive Medicine, National Center for Child Health and Development, Tokyo, Japan; 2 Department of Health Policy, National Center for Child Health and Development, Tokyo, Japan; 3 Division of Biostatistics, National Center for Child Health and Development, Tokyo, Japan; 4 Department of Orthopaedic Surgery, Shinshu University school of Medicine, Matsumoto, Japan

**Keywords:** Fracture, Osteoporosis, Preconception care, Pregnancy, Underweight

## Abstract

**Background:**

Undernutrition and underweight are osteoporosis risk factors. Therefore, improving the health of underweight young women in Japan is an important medical issue. However, few studies have evaluated the association between being preconception underweight and postnatal osteoporotic fractures in young women.

**Methods:**

This retrospective cohort study used a Japanese nationwide claims database (JMDC Inc.) to evaluate the effect of preconception underweight on the incidence of osteoporotic fracture within two years after delivery. Data from 16,684 mothers who delivered their first singleton babies between January 2006 and December 2020 were analysed. The combination of disease codes of fractures at sites associated with osteoporosis and medical procedures for fractures was defined as the incidence of osteoporotic fractures, whereas the body mass index (BMI) recorded 12–36 months before delivery was used as the exposure. We estimated the incidence of osteoporotic fractures by BMI category using a Kaplan–Meier curve and examined the fracture risk using Cox hazard regression analyses.

**Results:**

Fifty-one women (0.31%) were affected by osteoporotic fractures within two years of delivery. More than 80% of these were rib fractures, and approximately 65% of fractures occurred after the first year postpartum. Preconception underweight (BMI < 18.5 kg/m^2^) was significantly associated with the incidence of postpartum osteoporotic fractures. There was no significant association between low BMI and postnatal fractures, as analysed via multiple categorical logistic regression analysis.

**Conclusion:**

Appropriate control of preconception weight might be critical to improving the postpartum quality of life, subsequent bone health, and neonatal care environment.

**Supplementary Information:**

The online version contains supplementary material available at 10.1186/s12884-024-06514-y.

## Background

In recent years, being underweight has become a major issue in women’s healthcare in Japan. Japan is one of the developed countries with the most advanced trend of underweight females [1, 2]. Women with a body mass index (BMI), which is used as a criterion for estimating underweight and undernutrition, of less than 18.5 kg/m^2^ account for 11.5% of all Japanese women, reaching as high as 20.7% among young women in their 20s [3]. The high prevalence of low body weight among Japanese young women is suggested to be a result of more women embracing the Western appearance culture and thin-ideal standard propagated by the media. A shift to collectivist cultures, characterized by conformity to social norms, can more substantially affect food behaviours and increase eating disorders [4]. The mean age of Japanese primipara pregnant women is 30.7 years [5], which coincides with the age of women with a high prevalence of low body weight. According to multiple studies, severe underweight among middle-aged women aged 40 years or older not only increases the risk of noncommunicable diseases, such as type 2 diabetes mellitus and chronic kidney disease, but also causes osteoporosis and the associated locomotive syndrome [6, 7]. Thus, similar risks are assumed to be present for young women aged less than 40 years. However, many previous studies examining the association between osteoporosis and being underweight have focused on women aged 60 years or above, and few studies have examined the impact of being underweight in females at the reproductive age of 20–40 years on future bone health.

Pregnancy- and lactation-associated osteoporosis (PLO), a rare type of osteoporosis that develops in women of reproductive age, develops during late pregnancy or breastfeeding and is characterized by osteoporosis-related fractures, mainly vertebral body fractures, with severe back pain [8, 9]. Although the prevalence is low at 0.04–0.08% [10], PLO greatly impairs the mental state and quality of life of postpartum women, as it develops at a time when women are busy with breastfeeding and childcare and imposes a huge burden [11, 12]. The aetiology of PLO remains unclear, but its onset involves women’s physical characteristics before pregnancy, such as underweight and undernutrition, as with normal osteoporosis [13], in addition to physical or endocrinological changes in women from late pregnancy to after delivery, such as calcium loss from the body due to breastfeeding, a postpartum drop in blood oestrogen, and an increase in blood parathyroid hormone-related proteins [14, 15]. Therefore, to clarify the impact of being underweight in women of reproductive age on their future bone health, we focused on the association between preconception underweight and postpartum osteoporosis-related fragile fractures.

In addition, we used the real-world data (RWD) accumulated in the Japanese Medical Data Center (JMDC) Claims Database (JMDC Inc., Japan) in this study. Retrospective cohort studies using RWD target many cases with various backgrounds; therefore, they allow causal assumptions even for diseases with a low prevalence. Furthermore, in the JMDC Claims Database, claims and specific health check-up results have been anonymized, as entrusted by multiple health insurance associations. The JMDC Claims Database includes a wide variety of claims data, such as medical inpatient claims, medical outpatient claims, and dispensing claims, allowing follow-ups of outpatient visits and cases where consultation has been sought at multiple medical institutions. As many fracture treatments are provided as outpatient treatments, considering the reality of fracture treatment in Japan, the JMDC Claims Database was considered the most suitable RWD for this study.

Therefore, to explore the association between preconception underweight and postpartum fragility fractures in young Japanese women, we conducted a retrospective cohort study using the JMDC Claims Database.

## Methods

We performed a retrospective observational analysis using the JMDC Claims Database (JMDC, Inc., Japan). The JMDC database is an epidemiological claims database that accumulates claims (inpatient, outpatient, and dispensing) and health check-up data received from multiple health insurance associations and mainly includes the data of employees and their families of relatively large companies in Japan. The JMDC Claims Database is used to understand the actual state of various diseases and medical treatments, and the details of the database have already been described in other papers [16–18]. The study was conducted in accordance with the tenets of the Declaration of Helsinki. In addition, we obtained approval from the Ethics Committee of the National Center for Child Health and Development before conducting this study (approval number 2021 − 157). We did not obtain informed consent because we used only an anonymized aggregate dataset. In this study, we used the JMDC Claims Database from January 2006 to December 2020 and extracted two datasets, namely, the Children and Women datasets. First, for the children’s dataset, out of the children born, we extracted data of children whose “birth date” matched the “insurance subscription date.” Next, for the women’s dataset, out of the women linked to babies by the family ID, we extracted data of women who were in an “own person” or “spouse” relationship with the subscriber. We combined these two datasets with household flags to create a mother-child dataset.

**Fig. 1:**
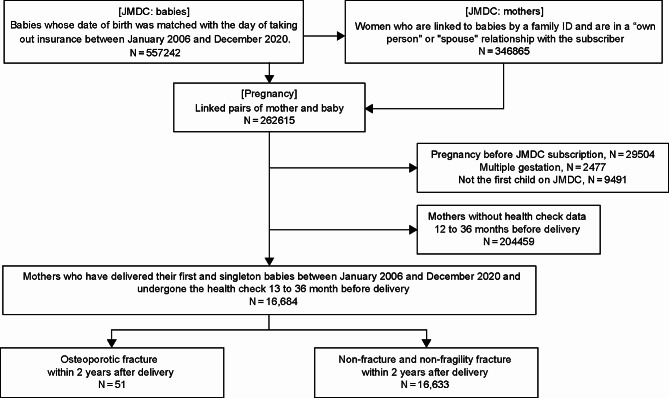
Flowchart of the study. Osteoporotic fracture was defined as vertebral fractures, distal radius fractures, proximal humerus fractures, femoral proximal fractures, rib fractures, pelvic fractures, and lower leg fractures. JMDC, Japanese nationwide claims database

From the mother-child dataset, we excluded mother-child pairs with the child’s birth date after the mother’s insurance subscription period (*n* = 29,504), mother-child pairs with multiple gestations (*n* = 2,477), mother-child pairs with older siblings (*n* = 9,491), and mother-child pairs without regular maternal health check-up data 12–36 months before the child’s birth date (*n* = 204,459). Finally, 16,684 mother-child pairs were included in the analysis. We defined the child’s “birth date” as the delivery month for the women (Fig. 1).

### Body mass index

**Fig. 2:**
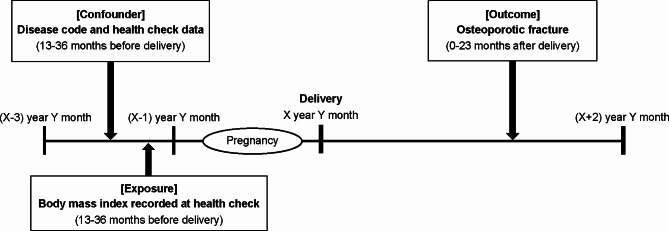
Study design. The combination of disease codes of closed osteoporotic fractures and medical procedures for fractures 0–23 months after delivery were defined as “occurring of osteoporotic fractures.” The disease code and the health check-up data recorded 12–36 months before delivery were used as confounders, whereas the body mass index recorded 12–36 months before delivery was used as the exposure in this study. The data recorded at the latest health check before delivery were used. However, the data during pregnancy were not used. If the latest data differed depending on the item in the medical check-up data, we used the data from the medical check-up when the body mass index was recorded at the nearest time before delivery

To define preconception underweight, we used BMI values included in the women’s health check-up data 12–36 months before the child’s birth date and divided the subjects into three groups according to BMI: <18.5 kg/m^2^, 18.5–24.9 kg/m^2^, and ≥ 25.0 kg/m^2^. The BMI was calculated as follows: weight (kg)/[height (m)]^2^. In cases where multiple health check-ups were performed 12–36 months before the child’s birth date and there were multiple BMI values, we adopted the data for the time point closest to the birth date (Fig. 2).

### Outcome

We targeted eight types of fragility fractures as the outcomes, namely lumbar, thoracic spine, proximal femoral, proximal humeral, distal radius, pelvic, lower leg, and rib fractures [19, 20], because these sites are considered easy to fracture with low-level or low-energy trauma if the bone is osteoporotic.

The subjects labelled with both the disease name code of closed fragility fractures and the medical procedure code within two years after delivery were included (Additional file 1: Table S1).

Disease name codes for open fractures were excluded because open fractures are often caused by a significant injury unrelated to osteoporosis. Patients labelled with the disease name code for apparent primary or secondary osteoporosis before pregnancy were excluded.

The apparent osteoporosis diseases were as follows: postmenopausal osteoporosis, primary or idiopathic osteoporosis, post-oophorectomy osteoporosis, disuse osteoporosis, postoperative malabsorption osteoporosis, steroid-induced osteoporosis, drug-induced osteoporosis, juvenile or geriatric osteoporosis, secondary osteoporosis, spinal osteoporosis, and neoplastic pathological fractures (Additional file 1: Table S1). Owing to the nature of the database, the medical treatment of traffic injuries was not included in the JMDC claims database.

### Measurements and definitions

We surveyed the mothers’ ages at delivery as well as the presence or absence of caesarean section (CS), diabetes mellitus, hyperlipidaemia, endocrine diseases, gynaecologic cancers, and anorexia nervosa, all of which may cause osteoporosis as a complication. These diseases were defined using injury/disease names or medical procedure codes (Additional file 1: Table S2). We extracted data on smoking, alcohol intake, and laboratory findings from the health check-up data. For the laboratory findings, we defined a haemoglobin concentration of less than 12 g/dL as anaemia, a total cholesterol level of less than 128 mg/dL as undernutrition, and an estimated glomerular filtration rate (eGFR) of less than 60 mL/min as an indicator of chronic kidney disease. The formulas were as follows: total cholesterol level = (serum low-density lipoprotein cholesterol) + (serum high-density lipoprotein cholesterol) + (serum total glycerides/5) and eGFR = 194 × (serum creatinine^− 1.094^) × (age^− 0.287^) × 0.739.

### Statistical analysis

First, we described the incidence and timing of fractures within a period of up to two years after delivery and the types of fractures. Next, we classified the subjects according to age; preconception BMI; complications reported to be associated with osteoporotic fractures, such as alcohol intake, smoking, anaemia, undernutrition, chronic kidney diseases, diabetes mellitus, endocrine diseases, malignant tumours (gynaecologic cancers and breast cancer), and the use of proton pump inhibitors and compared the incidence of osteoporotic fractures in each group.

For the use of proton pump inhibitors, we used the disease name of reflux esophagitis, for which they were used as standard therapeutic drugs. In addition, to examine the impact of the delivery mode on fractures, we classified the subjects by the presence or absence of CS and compared the fracture rates. Subsequently, we showed the cumulative incidence of fractures within two clinical postpartum years in each group using Kaplan–Meier curves.

Then, as the incidence of fractures associated with the obesity group (≥ 25.0 kg/m^2^) was low, we classified the subjects into two groups of underweight (BMI < 18.5 kg/m^2^) and above standard (BMI ≥ 18.5 kg/m^2^) by their BMI. Finally, to explore the factors affecting the survival data of osteoporotic fractures, we calculated the crude hazard ratio and adjusted hazard ratio, as well as the 95% confidence interval, using the Cox proportional hazards model, with the presence or absence of osteoporotic fractures as the dependent variable. The age at delivery, BMI, alcohol intake, smoking, laboratory findings, and complications were the independent variables, selected from the database with subjects with three or more fractures in each category when the variable was classified by category and with an intergroup difference of 0.1% or above in the fracture rate. We set smoking as an independent variable because it is considered a clear osteoporosis-related factor [21], even though a difference of only 0.3% was observed in the fracture rate between the categories. We used SAS software (version 15.0; SAS Institute, Cary, NC, USA) for the analyses performed in this study and set the significance level as 5%.

## Results

### Characteristics of women with fragility fractures within 2 years after delivery

Of the 16,684 subjects analysed, 51 (0.31%) developed fragility fractures within two years of delivery. Table 1 shows the time and site of the fracture in the fracture cases. For the time of fracture, most fractures occurred 12–14 months after delivery (11 cases), followed by 21–23 months after delivery (10 cases) and 18–20 months after delivery (9 cases). Of all cases, 64.7% (33 cases) experienced fractures 1–2 years after delivery.

**Table 1:** Characteristic of women with osteoporotic fractures within 2 years after delivery

Characteristics	Osteoporotic fracture (*N* = 51)	(%)
**Time of fracture occurring after delivery (months)**		
0	4	7.8
1–2	3	5.9
3–5	2	3.9
6–8	5	9.8
9–11	4	7.8
12–14	11	21.6
15–17	3	5.9
18–20	9	17.7
21–23	10	19.6
**Site of osteoporotic fractures**		
Vertebra		
Thoracic spine	0	0.0
Lumber spine	2	3.9
Distal radius	2	3.9
Proximal humerus	0	0.0
Proximal femoral	0	0.0
Ribs	44	86.3
Pelvic fracture	0	0.0
Lower leg	3	5.9

For the site of fracture, rib fractures were the most common, accounting for 86.3% of all fracture cases. The other fractures involved the lumbar spine in 2 cases (3.9%), distal radius in 2 cases (3.9%), and lower leg in 3 cases (5.9%). While both cases of lumbar spine fractures occurred within two months after delivery, distal radius fractures and lower leg fractures occurred 15 months after delivery (lumbar spine fracture: 1–2 months after delivery: 2 cases; distal radius fracture: 15–17 months after delivery: 1 case, 18–20 months after delivery: 1 case; lower leg fracture: 18–20 months after delivery: 2 cases, 21–23 months after delivery: 1 case).

### Incidence of osteoporotic fractures of the subjects between clinical categories

In Table 2, we compare the incidence of osteoporotic fractures in the subjects between the clinical categories assigned based on age, BMI, smoking and alcohol consumption, mode of delivery, complications, and several laboratory measurements recorded during the health checks. Women over 40 and aged 30–40 years showed a higher incidence of osteoporotic fractures than those aged 20–30 years (0.37% and 0.31%, respectively, vs. 0.18%). With regard to the BMI, the prevalence of fractures among women with a low BMI (< 18.5 kg/m^2^) was higher than that among normal (18.5–24.9 kg/m^2^) or heavy (> 25 kg/m^2^) BMI subjects (0.47% vs. 0.25% and 0.21%, respectively). Women who underwent CS and drank alcohol every day also seemed to have fractures more frequently than those who did not. The fracture incidence was similar between the groups when the assignation was based on smoking habits, laboratory data recorded during the health check, or complications.

**Table 2:** Incidence of osteoporotic fractures of the subjects between clinical categories

	All (*N* = 16,684)	(%)	Osteoporotic fracture cases (*N* = 51)	(%)	Non-fracture or non-Osteoporotic fracture cases (*N* = 16,633)	(%)
Age at delivery† (years)						
Over 40	2936	17.60	11	0.37	2925	99.63
30 to less than 40	11,490	68.87	36	0.31	11,454	99.69
20 to less than 30	2255	13.52	4	0.18	2251	99.82
Less than 20	0	0.00	0	0.00	0	0.00
BMI‡ (kg/m^2^)						
Low (less than 18.5)	3393	20.34	16	0.47	3377	99.53
Normal (18.5–24.9)	11,405	68.36	29	0.25	11,376	99.75
Heavy (over 25)	1444	8.65	3	0.21	1441	99.79
Alcohol intake*						
Every day	923	5.53	4	0.43	919	99.57
Not every day	12,823	76.86	35	0.27	12,788	99.73
Smoking⁑						
Habitual	1161	6.96	3	0.26	1158	99.74
Not habitual	13,468	80.72	39	0.29	13,429	99.71
Delivery mode						
Caesarean section	4094	24.54	18	0.44	4076	99.56
Others	12,590	75.46	33	0.26	12,557	99.74
Laboratory findings at the health check						
Haemoglobin level (g/dL) ⁂						
less than 12	1739	10.42	5	0.29	1734	99.71
more than 12	11,985	71.84	39	0.33	11,946	99.67
Serum T.chol level (mg/dL) ¶						
less than 128	188	1.13	1	0.53	187	99.47
more than 128	14,790	88.65	43	0.29	14,747	99.71
eGFR (mL/min) §						
less than 60	54	0.32	1	1.85	53	98.15
more than 60	9081	54.43	26	0.29	9055	99.71
Complications						
Diabetes mellitus						
yes	389	2.33	1	0.26	388	99.74
no	16,295	97.67	50	0.31	16,245	99.69
Hypertension						
yes	179	1.07	1	0.56	178	99.44
no	16,505	98.93	50	0.30	16,455	99.70
Hyperlipidaemia						
yes	563	3.37	2	0.36	561	99.64
no	16,121	96.63	49	0.30	16,072	99.70
Hyperparathyroidism						
yes	8	0.05	0	0.00	8	100.00
no	16,625	99.69	51	0.31	16,625	99.69
Hyperthyroidism						
yes	281	1.68	0	0.00	281	100.00
no	16,403	98.32	51	0.31	16,352	99.69
Hypogonadism						
yes	5605	33.60	18	0.32	5587	99.68
no	11,079	66.40	33	0.30	11,046	99.70
Anorexia nervosa						
yes	22	0.13	1	4.55	21	95.45
no	16,662	99.87	50	0.30	16,612	99.70
Reflux esophagitis						
yes	1103	6.61	4	0.36	1099	99.64
no	15,581	93.39	47	0.30	15,534	99.70
Gynaecologic malignancies and breast cancer						
yes	60	0.36	0	0.00	60	100.00
no	16,624	99.64	51	0.31	16,573	99.69

BMI, body mass index; eGFR, estimated glomerular filtration rate; Hb, haemoglobin; T.chol, total cholesterol; †ages were missed in 3 unfractured cases; ‡ body mass index was missed in 3 fracture cases and in 439 unfractured cases; ⁑ information about smoking was missed in 9 fracture cases and in 2,046 unfractured cases; habitual smoking is defined as a smoking habit of a total of 100 or more cigarettes or smoking for more than 6 months at the time of the health check; * information about alcohol was missed in 12 fracture cases and in 2,926 unfractured cases; ⁂ information about laboratory findings of the haemoglobin level was missed in 7 fracture cases and in 2,953 unfractured cases; ¶ information about laboratory findings of the serum total cholesterol level was missed in 7 fracture cases and in 1,699 unfractured cases; § information about the estimated glomerular filtration rate was missed in 24 fracture cases and in 7,525 unfractured cases

### Cumulative incidence of osteoporotic fracture within two years after delivery

**Fig. 3:**
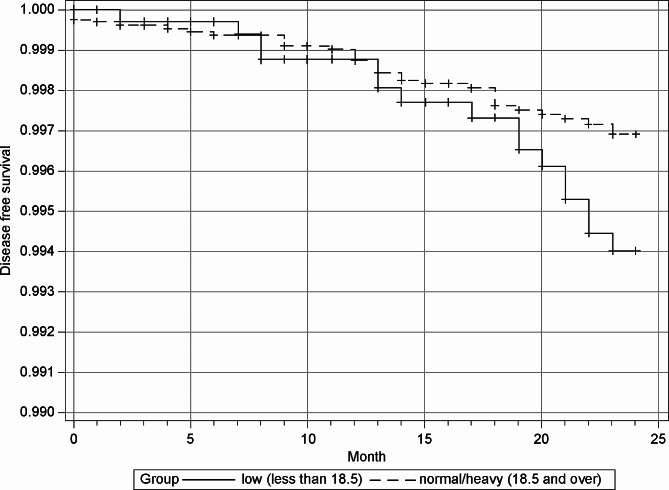
Kaplan–Meier survival: cumulative incidence of osteoporotic fractures in women within 2 years post-delivery. Low body weight, the body mass index is less than 18.5 kg/m^2^ (solid line); more than normal body weight, the body mass index is over 18.5 kg/m^2^ (dotted line). Group — underweight (BMI < 18.5 kg/m2, solid line), -– normal/ heavy weight (BMI ≥18.5 kg/m^2^, dotted line)

Figure 3 shows the Kaplan–Meier estimates of the cumulative incidence of osteoporotic fractures among women with a low BMI and those with a normal or heavy BMI. The cumulative incidence of osteoporotic fractures was similar between the two groups up to one year after delivery; however, after one year, the fracture rate increased in the low BMI group, exceeding the cumulative incidence in the normal and heavy BMI groups.

### Factors associated with osteoporotic fractures occurring within two years after delivery

Women with a low BMI show significantly higher ratio of osteoporotic fractures within two years after delivery than those with a normal or heavy BMI (hazard ratio: 1.8, 95% confidence interval: 1.01–3.34) (Table 3). However, there was no significant association between a low BMI and postnatal osteoporotic fractures on multiple categorical logistic regression analysis using the age at delivery, daily alcohol intake, habitual smoking, and CS as adjusted variables. Other factors were not correlated with the incidence of osteoporotic fractures within two years of delivery, even in the crude analysis.

**Table 3:** Factors associated with osteoporotic fractures occurring within 2 years after delivery

	HR	(95% CI)	*P*-value	aHR†	(95% CI)	*P*-value
Age at delivery						
20 to less than 30	Ref			Ref		
30 to less than 40	1.7	(0.62, 4.86)	0.2992	1.1	(0.36, 3.04)	0.9287
Over 40	2.0	(0.65, 6.43)	0.2198	0.9	(0.27, 3.27)	0.9169
BMI						
Over 18.5	Ref			Ref		
Less than 18.5	1.8	(1.01, 3.34)	0.048‡	1.3	(0.60, 2.73)	0.5295
Alcohol intake everyday (ref: No)	1.6	(0.58, 4.60)	0.3523	1.9	(0.65, 5.32)	0.2488
Habitual smoking (ref: No)	0.9	(0.28, 2.92)	0.8641	0.6	(0.15, 2.68)	0.5348
Caesarean section (ref: No)	1.7	(0.94, 2.97)	0.0802	1.5	(0.75, 308)	0.2440

aHR, adjusted hazard ratio; BMI, body mass index; HR, hazard ratio; Ref, reference; CI, confidence interval. † Adjusted HR and 95% CIs were calculated by multiple categorical logistic regression analysis using the age at delivery, BMI, alcohol intake every day, habitual smoking, and caesarean section as adjusted variables. ‡ statistically significant

## Discussion

Our retrospective cohort study using the JMDC database revealed the potential association between pre-conception underweight status and the incidence of osteoporotic fractures within two years after delivery. In total, 0.31% of all postpartum women developed osteoporotic fractures within two years after delivery, more than 80% of them were rib fractures, and approximately 65% developed fractures after one year after delivery. Preconception underweight (BMI < 18.5 kg/m^2^) was significantly associated with the incidence of postpartum fragility fractures. However, a significant association was not observed between a low preconception BMI and postnatal fractures in multiple categorical logistic regression analysis using the age at delivery, daily alcohol intake, habitual smoking, and delivery mode as adjusted variables.

The frequency, common site, and time of osteoporotic fractures shown in our study differ from the previously reported PLO characteristics. Although epidemiological information on PLO is limited, based on previous studies, the incidence of PLO is approximately 4–8 per 1 million people [9], the most common site is the vertebral body, and it most frequently occurs from the 3rd trimester of gestation to three months after delivery [10]. In contrast, in our study, the incidence of osteoporotic fractures within two years after delivery was 0.31%, which is approximately 1,000 times higher than that reported previously. In addition, spinal fractures accounted for approximately 4% of the total, and the fracture cases within three months after delivery only occurred in approximately one out of seven women. Such deviations could be because of the differences in research methods of our study compared with those of previous studies on PLO.

Most case reports or series and retrospective cohort studies on PLO have defined the disease based on clinical symptoms and the bone density [22]. On the other hand, we extracted data on osteoporotic fractures, a clinical outcome related to osteoporosis, by combining the disease name code of closed fractures in sites associated with osteoporosis and the medical procedures from the claims data of more than 10,000 women.

In a previous study that examined the fracture rate within two years of obstetric hospitalization using RWD, the incidence of osteoporotic fractures within two years after delivery was 0.045%, which was approximately 10% of the incidence calculated in our study [23]. This showed that there may have been cases other than the narrowly defined PLO among the extracted subjects. Because we conducted the study by further expanding the range to women without a history of obstetric hospitalization and women who underwent fracture treatment as outpatients, we may have had a broader spectrum of postnatal fragility fractures other than those related to PLO. However, fractures can negatively impact the postpartum quality of life, mental status, and work ability in women [11, 12]. As our study shows that more women suffer from osteoporotic fractures at a time imposed with postpartum burdens and require medical and social support than previously reported, we believe that our results could provide new important sights for the area of perinatal female bone healthcare.

Additionally, focusing on the sites of fracture, more than 80% of osteoporotic fractures within two years after delivery represented in this study were rib fractures. Although some rib fractures may be caused by trauma, the JMDC Claims Database does not include the medical procedure code for injuries caused by traffic accidents. Furthermore, we excluded subjects labelled with the disease code of open fracture, as women within two years after delivery are unlikely to perform high-intensity exercises that may induce fractures. Therefore, rib fractures are considered osteoporotic fractures that occur in the setting of low-level or low-energy trauma.

A history of fracture is a risk factor for future fractures [24] and is known to be significantly correlated with increased mortality risk in older adults, even if they are non-hip, non-vertebral fractures, including rib fractures [25]. The rib fracture, the major fracture among postpartum women in our study, has recently been reported as a predictor of future fractures in young and older postmenopausal women [26]. Therefore, viewing postpartum osteoporotic fractures as sentinel fractures may be necessary, which may increase the risk of future major fractures. For noncommunicable diseases such as hypertension and diabetes mellitus, pregnancy is known to be a window that allows a glimpse into future maternal health and a good opportunity for implementing appropriate preventive interventions [27]. For women who develop osteoporotic fractures after delivery, appropriate vitamin D supplementation and calcium intake, guidance of exercise habits, regular bone density measurement, and drug treatment may prevent future major fractures and extend their healthy life expectancy.

This study had several limitations. First, information regarding breastfeeding, physical activity, vitamin D insufficiency status, and family history, which are among the major causes of postpartum osteoporotic fractures, was not included in the analysis. However, to examine the incident risk of postpartum fracture, which is a rare disease, we believe that the advantage of being able to conduct the study using a database of many cases, such as the JMDC database, outweighs the disadvantage of not being able to examine detailed confounders. Second, as the incidence of fractures in the high BMI group was low, factors affecting the survival data of osteoporotic fractures could not be examined between the low BMI group (< 18.5 kg/m^2^) and the normal or high BMI group (≥ 18.5 kg/m^2^). In recent years, the increase in the number of young obese women has also become an issue in Japan. Thus, examining the risks for thin women with normal weight and obese women by conducting studies several years later after collecting sufficient data may be interesting. Third, we were unable to obtain information about the bone mineral density and bone metabolism markers from the database. These data are essential for the diagnosis of osteoporotic fractures. To overcome this limitation, we attempted to define osteoporotic fractures by combining medical procedures and disease name codes of closed fractures at eight osteoporosis-related bone sites based on expert opinion. Furthermore, we excluded the case of a major traffic injury and apparent primary or secondary osteoporosis before pregnancy. Finally, we used only one definition to obtain data on fragility fractures from the database. In the future, using other definitions would be worthy to examine the validity of the definition of postpartum fragility fracture.

## Conclusions

We examined the correlation between preconception underweight (BMI < 18.5 kg/m^2^) and osteoporotic fractures within two years after delivery using an RWD in Japan. Although preconception underweight was significantly associated with the incidence of postpartum osteoporotic fractures, no significant association was shown using multiple categorical logistic regression analysis. Proper weight management for preconception women might be essential to improve the postpartum quality of life, prevent future fractures, and improve the bone health of the next generation.

### Electronic supplementary material

Below is the link to the electronic supplementary material.

Supplementary Material 1

## Data Availability

The data supporting the findings of this study are available from the corresponding author, Kayoko Kaneko, upon reasonable request.

## Abbreviations

BMI: Body mass index
CS: Caesarean section
eGFR: Estimated glomerular filtration rate
JMDC: Japanese Medical Data Center
PLO: Pregnancy- and lactation-associated osteoporosis
RWD: Real-world data
