# Supplementary material for: Preconception underweight impact on postnatal osteoporotic fracture: a retrospective cohort study using Japanese claims data
Source: BMC Pregnancy Childbirth. 2024 Apr 25;24:315. doi: 10.1186/s12884-024-06514-y (PMC11044344; doi:10.1186/s12884-024-06514-y)
Supplement: Supplementary file 1 — Supplementary Material 1 [file 12884_2024_6514_MOESM1_ESM.docx]

***Original Article***

**Preconception underweight impact on postnatal osteoporotic fracture: A retrospective cohort study using Japanese claims data**

Kayoko Kaneko^1,*^, Maiko Suto^2^, Eiko Miyagawa^1^, Masashi Mikami^3^, Yukio Nakamura^4^, Atsuko Murashima^1^, Kenji Takehara^2^

^1^Division of Maternal Medicine, Center for Maternal-Fetal, Neonatal and Reproductive Medicine, National Center for Child Health and Development, Tokyo, Japan

^2^Department of Health Policy, National Center for Child Health and Development, Tokyo, Japan

^3^Division of Biostatistics, National Center for Child Health and Development, Tokyo, Japan

^4^Department of Orthopaedic Surgery, Shinshu University school of Medicine, Matsumoto, Japan

^*^***Corresponding author:*** Kayoko Kaneko, M.D.

Division of Maternal Medicine, Center of Maternal-Fetal, Neonatal and Reproductive Medicine, National Center for Child Health and Development

2-10-1, Okura, Setagaya-ku, 157-8535, Tokyo, Japan

Phone: 81-3-3416-0181

E-mail: [kaneko-ky@ncchd.go.jp](mailto:kaneko-ky@ncchd.go.jp)

**Table S1.** Disease and surgical procedure codes to identify fragility fractures

| Disease name | Disease Code | Procedure Codes |
| --- | --- | --- |
| **[Inclusion criteria]** | | |
| Lumbar fracture/closed | 8054003, 8840814, 8840815, 8054016, 8054007, 8844924, 8844926, 8842635, 8849001, 8840825, 8840814, 8840815, 8054016, 8054007, 8844924, 8844926, 8842635, 8849001, 8840825 | 140048350 |
| Thoracic spine fracture/closed | 8844858, 8832545, 8844853, 8842484, 8052003, 8832552, 8842568, 8848866, 8845886, 8845535, 8832545, 8844853, 8842484, 8052003, 8832552, 8842568, 8848866, 8845886, 8845535 | 140048450 |
| Proximal femoral fracture/closed | 8208003, 8208004, 8833731, 8208015, 8847522, 8837297, 8837298, 8837299, 8837300, 8208009, 8837303, 8837305, 8837306, 8837311, 8837314, 8837382, 8837384, 8837944, 8837945, 8208004, 8833731, 8208015, 8847522, 8837297, 8837298, 8837299, 8837300, 8208009, 8837303, 8837305, 8837306, 8837311, 8837314, 8837382, 8837384, 8837944, 8837945 | 150016710, 150018310, 150019210 |
| Proximal humeral fracture | 8835500, 8847369, 8835505, 8835506, 8122002, 8120003, 8835510, 8120006, 8847498, 8835517, 8835518, 8847369, 8835505, 8835506, 8122002, 8120003, 8835510, 8120006, 8847498, 8835517, 8835518 | 150016610, 150018210, 150019110, 140040810 |
| Distal radius fracture | 8134017, 8833324, 8134002, 8842348, 8848088, 8838017, 8845136, 8838021, 8134018, 8833324, 8134002, 8842348, 8848088, 8838017, 8845136, 8838021, 8134018 | 150016810, 150018410, 150019310, 140040810 |
| Pelvic fracture | 8845558, 8835306, 8840288, 8845616, 8845617, 8845618, 8088001, 8847754, 8834126, 8834129, 8840803, 8835306, 8840288, 8845616, 8845617, 8845618, 8088001, 8847754, 8834126, 8834129, 8840803 | 150060410, 150060910 |
| Lower leg fracture | 8832890, 8832891, 8832892, 8832893, 8832895, 8847464, 8832898, 8848869, 8832899, 8832904, 8230036, 8832906, 8832909, 8846262, 8846263, 8843712, 8843745, 8232005, 8230014, 8846266, 8846267, 8839229, 8848870, 8832911, 8842342, 8832889, 8844041, 8846258, 8846259, 8839223, 8839224, 8839225, 8839226, 8230026, 8847550, 8839228, 8232006, 8230027, 8230030, 8831310, 8832888, 8847062, 8230017, 8832891, 8832892, 8832893, 8832895, 8847464, 8832898, 8848869, 8832899, 8832904, 8230036, 8832906, 8832909, 8846262, 8846263, 8843712, 8843745, 8232005, 8230014, 8846266, 8846267, 8839229, 8848870, 8832911, 8842342, 8832889, 8844041, 8846258, 8846259, 8839223, 8839224, 8839225, 8839226, 8230026, 8847550, 8839228, 8232006, 8230027, 8230030, 8831310, 8832888, 8847062, 8230017 | 150016910, 150018510, 150019410, 140040810 |
| Rib fracture | 8070011, 8070006, 8849009, 8070010, 8070005, 8070007, 8070009, 8070006, 8849009, 8070010, 8070005, 8070007, 8070009 | 150017450, 150123010 |
| **[Exclusion criteria]** | | |
| Osteoporosis (postmenopausal) | 8850326, 8844319, 8850327, 8850328, 8844320, 8844321, 8844322, 8844323, 8844324, 8850329 |  |
| Osteoporosis (primary) | 8849529, 8850185, 8844204, 8850186, 8850187, 8844205, 8844206, 8844207, 8844208, 8844209 |  |
| Osteoporosis (post-oophorectomy) | 8850348, 8850349, 8850350, 8850351, 8850352, 8850353, 8850354, 8850355, 8844338, 8850356 |  |
| Osteoporosis (disuse) | 8850308, 8850309, 8850310, 8850311, 8850312, 8850313, 8850314, 8850315, 8844313, 8850316 |  |
| Osteoporosis (postoperative malabsorption) | 8850223, 8850224, 8850225, 8850226, 8850227, 8850228, 8850229, 8850230, 8844238, 8850231 |  |
| Osteoporosis (steroid-induced) | 8850236, 8850237, 8850238, 8850239, 8850240, 8850241, 8850242, 8850243, 8844272, 8850244 |  |
| Osteoporosis (drug-induced) | 8850334, 8850335, 8850336, 8850337, 8850338, 8850339, 8850340, 8850341, 8844332, 8850342 |  |
| Osteoporosis (juvenile) | 8850214, 8850215, 8850216, 8850217, 8850218, 8850219, 8850220, 8850221, 8844237, 8850222 |  |
| Osteoporosis (idiopathic) | 8850287, 8850288, 8850289, 8850290, 8850291, 8850292, 8850293, 8850294, 8844303, 8850295 |  |
| Osteoporosis (geriatric) | 8850363, 8848170, 8850364, 8850365, 8848171, 8848172, 8848173, 8844345, 8850366 |  |
| Spinal osteoporosis | 8844274 |  |
| Osteoporosis (secondary) | 8850296, 8850297, 8850298, 8850299, 8850300, 8850301, 8850302, 8850303, 8844308, 8850304 |  |
| Osteoporosis (other than secondary) | 8849536, 7330028, 8840913, 8838912, 8835331, 7330026, 8840727, 8848091, 7330023, 8844302 |  |
| Neoplastic pathological fracture | 8849333, 8849335, 8849336, 8849337, 8849338, 8849339, 8849340, 8849341, 8849342, 8849343 |  |

**Table S2.** Disease and surgical procedure codes to identify fractures related to osteoporosis

| Disease name | Disease Codes | Surgical Procedure Codes |
| --- | --- | --- |
| Anorexia nervosa | 8830450, 3075011, 3075005, 8834947, 3075010, 8834950, 8834952, 7833001, 8839320, 8839321 |  |
| Reflux oesophagitis | 8845215, 8843825, 5301002, 8850989, 8844239, 8845322, 8838366 |  |
| Hypoparathyroidism | 2520001, 2271005, 2520010, 2520003, 8844594 |  |
| Hyperthyroidism | 2429001, 2429013, 2429012, 8844716, 8833502, 8833514, 8833515, 8833516, 8833517, 2420014, 8833519, 2429007, 2429015, 8841422 |  |
| Hypogonadism in females | 2563010, 8833321, 8833652, 8850477, 2563017, 8848077, 8842034, 2569009, 2569010, 8850918 |  |
| Uterine cancer | 1799003, 1985010, 1799004, 8846283, 8847757, 1800003, 1809004, 8848883, 8844722, 8842739, 1820002, 1820003, 8848716, 8834242, 8834243, 8847758, 1799007, 1820005, 1799005, 1799006, 8848717, 8845443 |  |
| Breast cancer | 8849699, 8848690, 8848843, 8830917, 8842759, 8848722, 1749004, 8848743, 8848981, 1749008, 8849815, 1985025, 1749009, 8849816, 1982005, 1749011, 8849183, 8849184, 8848773, 8845450, 1749015, 8838464, 8838465, 8845451, 8848646, 8845025, 8838475, 8838476, 8848647, 8838483, 1749017, 8838489, 8845452 |  |
| Diabetes mellitus | 2500014, 8841685, 8841682, 8841680, 8841679, 8841683, 8841681, 8841687, 8841686, 8841688, 8841684, 8845043, 8845044, 8845045, 8845842, 8843105, 8843982, 8845046, 8845047, 8845048, 8845049, 8845050, 8845051, 8849056, 8830028, 8849557, 8845052, 8845053, 8845054, 8830030, 8845055, 8845056, 8845057, 8845058, 8830031, 8843983, 8843984, 8843985, 8843986, 8843987, 8843988, 8843989, 8845059, 8844626, 8845060, 8845061, 8845062, 8845063, 8845064, 8845065, 8845066, 8845067, 8830032, 8844346, 8845068, 8844627, 8845069, 8845070, 8845071, 8830033, 8844022, 8844023, 8844024, 8844025, 8844026, 8844027, 8844028, 8844029, 8844030, 8844031, 8844045, 8844536, 2500027, 2500015, 8841695, 8841692, 8841690, 8841689, 8841693, 8841691, 8841697, 8841696, 8841698, 8841694, 8845072, 8845073, 8845074, 8848108, 8843106, 8843990, 8845075, 8845076, 8845077, 8845078, 8845079, 8845080, 8849058, 8830040, 8849558, 8845081, 8845082, 8845083, 8830041, 8845084, 8845085, 8845086, 8845087, 8830042, 8843991, 8843992, 8843993, 8843994, 8843995, 8843996, 8843997, 8845088, 8844628, 8845089, 8845090, 8845091, 8845092, 8845093, 8845094, 8845095, 8845096, 8830043, 8844347, 8845097, 8844629, 8845098, 8845099, 8845100, 8830044, 8830045, 8830405, 2500001, 8835244, 8844537, 2500037, 8845198, 8849874, 8830756, 8843120, 8843121, 8843122, 8843123, 8843124, 8843125, 8843126, 8843127, 8843128, 8849585, 8849586, 8849587, 8849588, 8849589, 8849590, 8849591, 8849592, 8849593, 8849594, 2500024, 8843375, 8843376, 8843377, 8843378, 8843379, 8843380, 8843381, 8843382, 8843383, 2509003, 8843388, 8843389, 8843390, 8843391, 8843392, 8843393, 8843394, 8843395, 8843396, 2509004, 8843448, 8843449, 8843450, 8843451, 8843452, 8843453, 8843454, 8843455, 8843456, 8840710, 8843619, 8843620, 8843621, 8843622, 8843623, 8843624, 8843625, 8843626, 8843627, 8832747, 8845128, 2502004, 2504010, 2500013, 8843439, 8848632, 2504012, 8844089, 2507028, 2501002, 2501003, 2506006, 8838063, 8838064, 2507025, 8838065, 2505021, 8838066, 8849181, 2501005, 8838067, 2504004, 8838068, 2502006, 8838069, 8848768, 2505011, 8838070, 8838071, 2503005, 8850065, 2503007, 8844652, 8838072, 8838073, 8838074, 8838075, 2504005, 8838076, 8838077, 2506011, 8838078, 2504006, 2507029, 8844653, 8838079, 8838080, 2505018, 8848633, 8848634, 2504013 |  |
| Hypertension | 8830212, 8832479, 8848337, 8833421, 8842178, 8849300, 8842488, 8842089, 8842500, 4019016, 4019017, 8842094, 8840107, 8833422, 4029010, 8833426, 4039001, 4039033, 4039005, 4039006, 8833427, 4039036, 4039025, 4039026, 4039028, 5879003, 8833425, 8849279, 8835586, 8835605, 8835614, 8838336, 8838398, 8839689, 8849491 |  |
| Hyperlipidaemia | 8849518, 8830371, 8831264, 2720001, 8845523, 8845524, 8831269, 8831270, 8831271, 8831272, 8831273, 8831274, 8831275, 8850975, 8831286, 8833120, 8849528, 2724037, 8851069, 2724036, 8851070, 2723001, 2720004, 8833435, 2724007, 2721002, 8833663, 8833722, 8833881, 8844446, 2729002, 8849534, 2724023, 8848266, 2729003, 8849543, 8851146, 8851147, 8837852, 8842204, 8851149, 8837884, 2724031, 8840108, 2724012, 8848306, 8840506, 3335006, 8849849, 8849690, 8840985 |  |
| Caesarean section |  | 150222110, 150222210, 150390570 |
